# Chondroitinase C Selectively Degrades Chondroitin Sulfate Glycosaminoglycans that Inhibit Axonal Growth within the Endoneurium of Peripheral Nerve

**DOI:** 10.1371/journal.pone.0167682

**Published:** 2016-12-14

**Authors:** James B. Graham, David Muir

**Affiliations:** Department of Pediatrics, College of Medicine, University of Florida, Gainesville, Florida, United States of America; University of Patras, GREECE

## Abstract

The success of peripheral nerve regeneration is highly dependent on the regrowth of axons within the endoneurial basal lamina tubes that promote target-oriented pathfinding and appropriate reinnervation. Restoration of nerve continuity at this structural level after nerve transection injury by direct repair and nerve grafting remains a major surgical challenge. Recently, biological approaches that alter the balance of growth inhibitors and promoters in nerve have shown promise to improve appropriate axonal regeneration and recovery of peripheral nerve function. Chondroitin sulfate proteoglycans (CSPGs) are known inhibitors of axonal growth. This growth inhibition is mainly associated with a CSPG's glycosaminoglycan chains. Enzymatic degradation of these chains with chondroitinase eliminates this inhibitory activity and, when applied in vivo, can improve the outcome of nerve repair. To date, these encouraging findings were obtained with chondroitinase ABC (a pan-specific chondroitinase). The aim of this study was to examine the distribution of CSPG subtypes in rodent, rabbit, and human peripheral nerve and to test more selective biological enzymatic approaches to improve appropriate axonal growth within the endoneurium and minimize aberrant growth. Here we provide evidence that the endoneurium, but not the surrounding epineurium, is rich in CSPGs that have glycosaminoglycan chains readily degraded by chondroitinase C. Biochemical studies indicate that chondroitinase C has degradation specificity for 6-sulfated glycosaminoglycans found in peripheral nerve. We found that chondroitinase C degrades and inactivates inhibitory CSPGs within the endoneurium but not so much in the surrounding nerve compartments. Cryoculture bioassays (neurons grown on tissue sections) show that chondroitinase C selectively and significantly enhanced neuritic growth associated with the endoneurial basal laminae without changing growth-inhibiting properties of the surrounding epineurium. Interestingly, chondroitinase ABC treatment increased greatly the growth-promoting properties of the epineurial tissue whereas chondroitinase C had little effect. Our evidence indicates that chondroitinase C effectively degrades and inactivates inhibitory CSPGs present in the endoneurial Schwann cell basal lamina and does so more specifically than chondroitinase ABC. These findings are discussed in the context of improving nerve repair and regeneration and the growth-promoting properties of processed nerve allografts.

## Introduction

Decellularized peripheral nerve grafts have the ability to support axon regeneration and recovery of function. This is attributed to the potent growth-promoting extracellular matrix (ECM) components found within the endoneurium of nerve fascicles [1]. The endoneurium contains tightly packed cylindrical basal laminae comprised of components including perlecan, laminin-2, nidogen (entactin), and collagens [2]. Each basal lamina forms a continuous tube-like structure that encases an axon and Schwann cells through the entire length of the nerve. Basal lamina tubes persist after axotomy and distal nerve degeneration and provide a path for axonal regrowth and target reinnervation.

Inhibitory CSPGs are present throughout the ECM of the peripheral nerve that suppress and restrict axonal growth [1]. CSPGs consist of a core protein to which linear chondroitin sulfate (CS) glycosaminoglycan (GAG) sugar chains are attached to a common tetrasaccharide linkage region. Each CS GAG chain consists of repeating disaccharide subunits containing a glucuronic acid and an N-acetylgalactosamine in a β1–3 glycosidic bond (GlcA β1–3 NGalAc) which are linked together with a β1–4 glycosidic bond. Several CS subunits have been identified based on the carbon position of an attached sulfate group. CS-A contains a 4-carbon sulfate on the NGalAc unit while CS-C contains a 6-carbon sulfate. Dermatan sulfate (DS), formally known as CS-B, contains an epimerized 5-carbon of the GlcA unit to form iduronic acid (IdoA) and can contain a sulfate group on the 2-carbon position of the IdoA unit and the 4-carbon position on the NGalAc unit. CS-D and CS-E have two sulfate groups on the 2-carbon of GlcA and 6-carbon of NGalAc or 4-carbon and 6-carbon of the NGalAc respectively. The sulfation patterns of CS/DS GAG chains influence the inhibitory nature of CSPGs [3]. Furthermore, it is now appreciated that each core protein can contain GAG chains that consist of either one or a mixture of CS/DS subunits [4] [5]. It is the heterogeneity of CSPGs that have complicated the process of identifying and targeting those responsible for neurite inhibition.

Interestingly, inhibitory CSPGs are upregulated after nerve injury, despite the nerve's ability to support axonal growth [6] [7]. However, nerve injury results in degeneration of the distal segment and degenerated nerve has greater growth-promoting potential than normal nerve [8] [9] [10]. In particular, the growth-promoting properties of the Schwann cell basal lamina are selectively unmasked and preserved in degenerated nerve [10] [11]. In order to encourage axon regeneration associated with the basal laminae within the endoneurium, an ideal nerve graft would have the same properties; that is, eliminating the growth inhibitory activity of the endoneurial CSPGs while preserving and retaining the growth-promoting properties of the basal lamina. Furthermore, it would be desirable to retain the inhibitory CSPGs in the surrounding nerve sheaths to discourage regenerating axons from wandering outside the endoneurium and nerve fascicle. There have been numerous attempts to produce effective nerve grafts by various extraction and preservation processes to remove inhibitors of axon regeneration and reduce immunoreactivity [12] [13] [14] [15]. Some have enhanced the outcome of nerve grafting by targeted degradation of inhibitory CSPGs throughout the nerve graft with chondroitinase ABC (ChABC) [16] [17] [18]. Interestingly, complete degradation of all CSPG GAG chains may lead to uncontrolled axonal sprouting resulting in inappropriate reinnervation [19].

In light of the enhanced regeneration of axons following ChABC treatment, we sought to identify, selectively degrade and inactivate the inhibitory CSPGs located within the nerve fascicles with the goal of creating a nerve graft that encouraged fascicle-directed regeneration. This becomes clinically relevant when considering the large amount of interstitial tissue between fascicles of the multifasciculated human nerve. In this study, we investigated the distribution of particular inhibitory GAG chains and CSPG subtypes within multifasciculated peripheral nerves and their susceptibility to degradation and inactivation by a chondroitinase with greater specificity than the pan-specific ChABC. Here we provide evidence that the endoneurial compartment contains CSPGs with GAG chains that are more specifically degraded by chondroitinase C (ChC) and that ChC selectively enhances the axon growth-promoting properties of the endoneurial basal lamina.

## Methods and Materials

### Nerve tissues and immunolabeling

Normal nerves were collected from adult male Sprague Dawley rats (Charles River Laboratories) and New Zealand White female rabbits (Millbrook Farms, NY) immediately after euthanasia. The protocol to acquire and use animal nerve tissue was approved by the University of Florida Institutional Animal Care and Use Committee. Rats were housed in pairs at the University of Florida Animal Care Facilities under standard conditions with food and water ad libitum, and a 12-hour light cycle. Rats were deeply anesthetized with isoflurane and decapitated. Rabbits were group housed in pens at the University of Florida Animal Care Facilities with standard 12-hour light cycles, with ad libitum access to enrichment objects, food, and water. Rabbits were initially anesthetized with an intramuscular injection of ketamine (35mg/kg), xylazine (5mg/kg), and acetylpromazine (1mg/kg). This was followed by an intracardiac injection of sodium pentobarbital (Euthasol 5mg/kg). Death was confirmed by absence of cardiac function followed by thoracotomy. Human nerve was obtained from amputated limbs destined for disposal from previously consented surgeries. The protocol to acquire and use human nerve tissue was approved by the University of Florida Institutional Review Board (UF IRB #123–2004). No identifiable personal data or information was obtained or retained. All nerve samples were frozen and stored at -80°C, embedded in medium for cryosection or fixed in buffered 4% paraformaldehyde and processed for paraffin embedding.

Nerve samples were sectioned and processed for immunocytochemical analysis. A list of primary antibodies including antigen specificity is shown in Table 1. Primary antibodies were diluted in blocking buffer (phosphate buffered saline containing 10% normal serum) and incubated on tissue sections overnight at 4°C. Bound primary antibodies were labeled with secondary antibodies conjugated to various fluorophores or horseradish peroxidase. Digital images were captured and optimized for presentation.

**Table 1:** Antibodies and Characterizations.

Antibody (Reference)	Antigen/Epitope	Immunolabeling requirements
PcAb Perlecan [22] (sc-25848, Santa Cruz Biotechnology, Santa Cruz, CA, USA)	Perlecan, intact from EHS tumor	Fresh-frozen tissue only
McAb 12C5 [23] (Developmental Studies Hybridoma Bank, Iowa City, Iowa, USA)	Versican, Hyaluronate-binding region	Fresh-frozen tissue only
PcAb N-15 [24] (sc-22613, Santa Cruz Biotechnology, Santa Cruz, CA, USA)	Decorin, N-terminus	Fresh-frozen tissue only
McAb CS-56 [25]; [26] (C8035, Sigma Aldrich, St, Louis, MO, USA)	Chondroitin sulfate glycosaminoglycan chain; Chondroitin sulfate octasaccharides sequences: CS-CADC, CS-CADA, CS-AADC, CS-AADA	None
McAb 473-HD [27]; [26] (sc-101825, Santa Cruz Biotechnology, Santa Cruz, CA, USA)	DSD-1 chondroitin sulfate epitope; Chondroitin sulfate hexasaccharide sequences: CS-CAD, CS-DAA, CS-DAD	None
PcAb anti- laminin-1 [28] (NB300-144, Novus Biologicals, Littleton, CO, USA)	Laminin-1, whole protein	Pepsin pretreatment on formalin fixed tissue
McAb 5H2 [29] (MAB1922, Millipore, Temecula, CA, USA)	Laminin, alpha2 chain	Pepsin pretreatment on formalin fixed tissue
McAb 2E8 [30] (MAB1920, Millipore, Temecula, CA, USA)	Laminin, beta2 chain	Pepsin pretreatment on formalin fixed tissue
McAb C6S [31] (MAB2035, Millipore, Temecula, CA, USA)	Unsaturated 6-sulfated residual GAG unit after chondroitinase ABC, AC, or testicular hyaluronidase digested adult human cartilage proteoglycans	ChABC or ChC treatment
McAb C4S [31] (MAB2030, Millipore, Temecula, CA, USA)	Unsaturated 4-sulfated chondroitinase ABC digested mouse and human cartilage proteoglycans	ChABC treatment
McAb 1E3 [32] (MCA-1E3, Encor Biotechnology, Inc. Gainesville, FL, USA)	Growth associated protein 43	None

### Determining optimum conditions for ChC treatment of nerve grafts

To determine the optimum condition for complete degradation of CS-C from the nerve fascicles, a series of incubation experiments were performed. The guidelines for optimum ChC activation were adopted from Michelacci and Dietrich (1976) [20]. Three-cm segments of rabbit peroneal nerve were freeze/thawed several times in liquid nitrogen to render them acellular and increase tissue permeability for the enzyme treatments. ChC was diluted into a 50mM Tris Buffer (pH 8.0) at a final concentration of 1U, 4U or 8U per ml and the nerve segments were incubated for either 4, 8, 16 or 24 hours at 25°C. Nerve segments were rinsed several times in PBS and fixed in 4% paraformaldehyde. Nerve segments were processed and embedded in paraffin and subjected to anti-C6S and anti-C4S immunocytochemistry as previously described. Serial sections were also immunostained for undigested CSPG GAGs using CS-56 antibody. In order to determine complete digestion of GAG chains susceptible to ChC activity, a secondary treatment of ChABC (0.2U/ml in 50mM Tris buffer pH 8.0 and 50mM NaCl) was applied directly onto serial nerve tissue sections. ChABC will fully digest all CSPG GAGs in the tissue, fully revealing the C4S and C6S neoepitope and removing all the CS-56 antigens. Digital images were captured and a direct comparison was performed to determine the optimum condition for ChC to maximize C6S fluorescent signal and eliminate CS-56 immunoreactivity.

### Proteoglycan-enriched nerve extract

Forty New Zealand White rabbit sciatic nerves were collected and stored in Lactated Ringer’s Saline (LRS) at -80C°. The nerves were placed into liquid nitrogen for 20 minutes and finely minced over dry ice. The nerve mince was suspended in homogenization buffer (20mM Tris-HCl, pH 7.5, 50mM NaCl, Protease Inhibitor Cocktail [Sigma #P2714]) and pureed with a rotary tissue homogenizer. The homogenate was centrifuged at 11,000g for 20 minutes. The buoyant myelin layer was carefully aspirated and the supernatant was stored at 4°C. The pellet was resuspended in tissue extraction buffer (50mM Tris-HCl, pH 7.5, 4M Guanidine-HCl, 0.5% CHAPS, Protease Inhibitor Cocktail) and agitated for 2 hours at 4°C, centrifuged for 30 min at 17,000g, and the supernatant was removed and stored. The pellet was resuspended in fresh extraction buffer and agitated overnight at 4°C. The nerve extract was then centrifuged for 30 min at 17,000g and the supernatant was removed and stored. Both extraction and homogenized supernatants were pooled together and the guanidine was removed by dialysis. The extract was brought to 50mM Tris-HCl pH 8.0, 7M Urea, 0.2M NaCl, 0.1% CHAPS, 0.5% Triton X-100, and protease inhibitors added. A 20 ml column of diethylaminoethyl sepharose (DEAE) was equilibrated with the Urea Buffer and the nerve extract applied. The column was washed with Urea buffer with 0.25M NaCl and 1% Triton X-100 followed by a second wash without Triton X-100. Bound proteoglycans were eluted with Urea Buffer (without Triton X100) containing 1M NaCl. The proteoglycan-enriched fraction was concentrated by 100kD ultrafiltration and dialyzed to water.

### Western immunoblot analysis

Samples of the proteoglycan-enrich nerve extract were treated with ChABC (0.2U/ml, C3667, protease free, Sigma-Aldrich), ChC (4U/ml, C0954, Sigma-Aldrich), or vehicle control for 24 hours (incubated at 37°C for ChABC and 25°C for ChC and vehicle control). The samples were electrophoresed under reducing conditions on a 4–15% SDS-PAGE minigel (Bio-rad, Hercules, CA) and then electroblotted to nitrocellulose sheets. The blots were fixed (25% ethanol, 10% acetic acid), washed with PBS and blocked with 5% milk in 1% Triton X-100 in PBS. Primary antibodies were diluted in blocking buffer and incubated on the blots overnight. Bound antibodies were detected with secondary antibodies conjugated with HRP and developed by chemiluminescence.

### Cryoculture

Cryoculture is a tissue culture bioassay that recapitulates many aspects of nerve regeneration [21]. Cryoculture bioassays were performed as described previously [16]. Briefly, fresh frozen rabbit tibial nerves were sectioned and mounted onto coverslips (pre-coated with 3-aminopropyltrimethoxysilane to ensure adhesion). The nerve sections were treated with: i) ChABC diluted to 0.1U/ml in 50mM Tris buffer, pH 8.0 containing 50mM NaCl and incubated for 4 hours at 37°C; ii) ChC diluted to 4U/ml in 50mM Tris, pH 8.0 and incubated for 24 hours at 25°C; iii) Vehicle control using ChC buffer only (50mM Tris pH 8.0) and incubated for 24 hours at 25°C. Dorsal root ganglia (DRG) were removed from P2 neonatal mice (C57BL/6J mice, University of Florida Animal Care Services murine colony) and dissociated with collagenase XI followed by trypsin and trituration [11]. Cells were collected by centrifugation, resuspended in DMEM containing 10% serum and cultured for 2 hours, during which time most of the non-neuronal cells attached to the dish. The non-adherent DRG neurons were collected and resuspended in N2 media supplemented with ß-nerve growth factor (10ng/ml). Neurons (5,000 in 0.1 ml) were seeded directly onto the pretreated nerve tissue sections and cultured for 48 hours under standard culture conditions. The cryocultures were fixed with 70% methanol and immunolabeled for GAP-43 (Encor Biotech, Gainesville, FL, USA) to visualize the neurons and their processes. Photomicrographs were captured and neurite lengths measured by image analysis using ImagePro Plus (Media Cybernetics, Rockville, MD). Data points were reported as the mean length (μm) ± SEM.

### Statistical Analysis

All neurite length measurements were grouped and initially tested for significant differences between standard deviations of conditions to determine parametric distribution. The Kruskal-Wallis non-parametric ANOVA was determined appropriate for comparing axon lengths due to significant differences in the standard deviations. A Dunn’s Multiple Comparison post-hoc analysis was performed to determine which conditions represented a significant difference of the mean scores. Significance was predetermined as a p-value <0.05.

## Results

### Localization of proteoglycans relative to laminin in the peripheral nerve of various species

**Fig 1:**
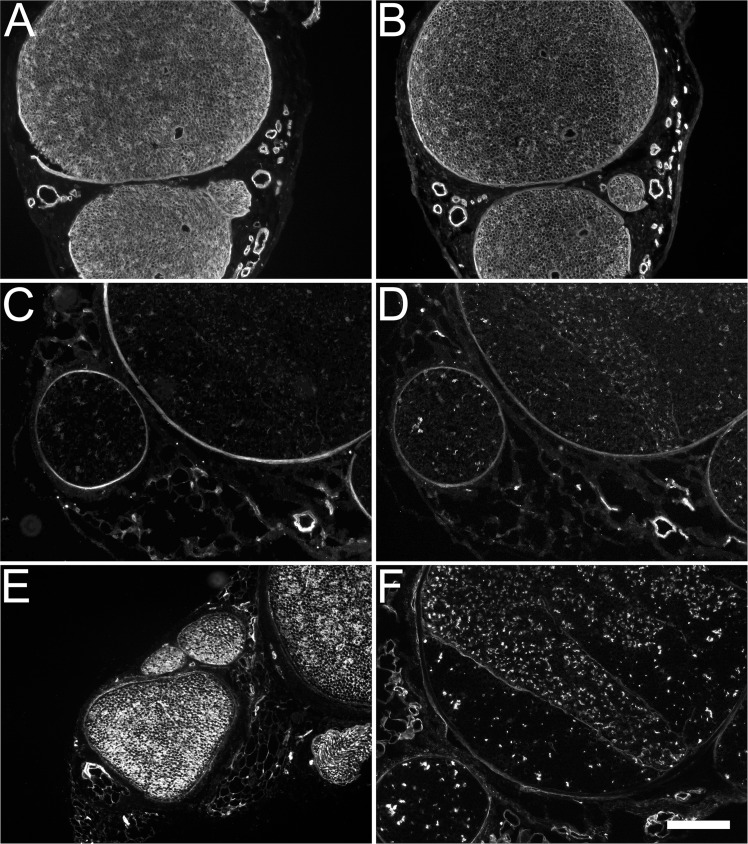
Distribution of proteoglycans relative to laminin in normal rat and rabbit peripheral nerve. Normal rat (A and B) and rabbit (C,D,E,F) sciatic nerve was subjected to immunohistochemical analysis with antibodies against laminin-2 (A), proteoglycan core proteins (B,C,D) and glycosaminoglycan chains (E and F). (A) Laminin was found within the nerve endoneurium and perineurium while only present within the basement membranes of blood vessels in the epineurium. (B) The heparan sulfate/chondroitin sulfate proteoglycan, perlecan, colocalized with laminin. (C) The chondroitin sulfate/dermatan sulfate proteoglycan, decorin, appeared primarily within the perineurium with faint immunoreactivity found amongst a subset of endoneurial tubes. (D) Versican (12C5) had a similar distribution to decorin primarily within the perineurium and faintly dispersed amongst a subset of endoneurial tubes. (E) A commonly used marker for chondroitin sulfate glycosaminoglycans, antibody CS-56, was mostly found within the endoneurium and appeared to label some components of the epineurium. (F) 473-HD, which has been reported to be specific for chondroitin sulfate/dermatan sulfate hybrid glycosaminoglycan chains, produced a similar staining pattern as decorin and versican. Scale bar: 100 μm.

Transverse sections of rat, rabbit, and human peripheral nerve were immunolabeled for several proteoglycan core proteins and GAGs (See Table 1 for antibody characterizations). In the rat, laminin-2 was present in the endoneurium and perineurium but absent from the epineurium (Fig 1A). Perlecan immunolabeling was intense throughout the endoneurium and perineurium (Fig 1B). This distribution was also observed within rabbit and human nerve fascicles (not shown). In the rabbit nerve, decorin was found exclusively in the perineurium surrounding each nerve fascicle (Fig 1C). Versican labeling of rabbit nerve was evident in the perineurium and also in a subset of small basal lamina clusters within the endoneurium (Fig 1D). The GAG antibody, CS-56, was strongly immunoreactive throughout the endoneurium and perineurium of the rabbit nerve (Fig 1E). The GAG antibody, 473HD, was isolated to a subset of small basal lamina clusters similar to the endoneurial distribution of versican in the rabbit nerve (Fig 1F).

### Distribution of ΔCS-A and ΔCS-C GAG in peripheral nerve following ChABC treatment

**Fig 2:**
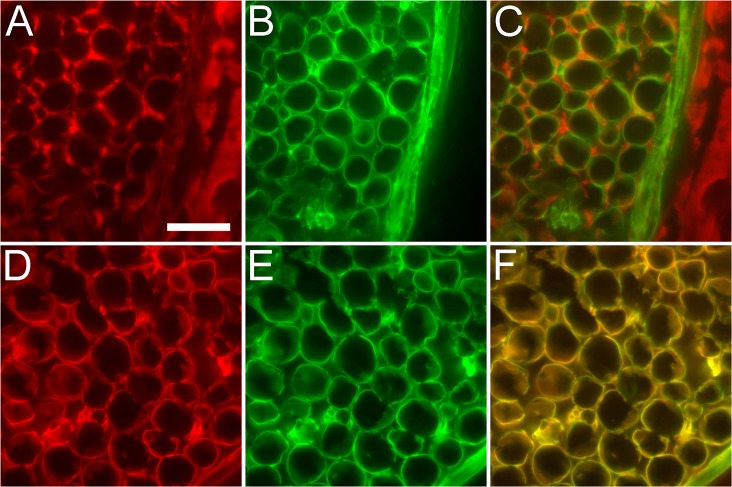
Endoneurial distribution of ΔCS-A and ΔCS-C relative to laminin in normal rat peripheral nerve. Normal rat sciatic nerve was subjected to chondroitinase ABC treatment and double immunolabeled with antibodies against laminin-1 and either ΔCS-A (C4S antibody) or ΔCS-C (C6S antibody). (A) C4S immunolabeling was present within the endoneurium but appeared to be isolated to the outside of the endoneurial tubes and seemed to be absent from the perineurium. (B) Immunolabeling for laminin revealed ring-like structures within the endoneurium that represent the boundaries of basal lamina tubes. Laminin was also present within the perineurium. (C) Combining both (A) and (B) provided a complementary pattern where the C4S (Red) immunoreactive areas were distinct and separate from the laminin (Green) immunoreactive rings of the basal lamina tubes. (D) C6S immunolabeling revealed a pattern that more resembled the ring-like laminin structures of the basal lamina (E). (F) Combining both (D) and (E) confirms that both components colocalize to the basal lamina tubes of the endoneurium. Scale bar: 20 μm.

ChABC degrades GAG chains by breaking the β1–4 glycosidic bond revealing an unsaturated residual GAG subunit (ΔCS) that is retained on the tetrasaccharide linkage region of CSPG core protein. Antibodies have been developed that recognize ΔCS based on the position of the sulfate group. C6S is very selective for the ΔCS-C while, C4S will recognize both ΔCS-A and the 4-sulfated ΔDS residual unit [28] [31]. Since the 4-sulfated ΔDS GAG unit loses its epimerized C-5 identity after ChABC treatment due to the double bond formed between C4 and C5 of the unsaturated hexuronic acid, we referred to both ΔCS-A and 4-sulfated ΔDS GAG units as ΔCS-A. Rabbit and rat nerve sections were treated with ChABC and immunolabeled with C4S and C6S antibodies to identify ΔCS-A and ΔCS-C antigens respectively. ΔCS-A was identified throughout most of the peripheral nerve ECM while ΔCS-C was only detected in the endoneurium as previously reported [1]. Double-labeling with ΔCS antibodies and anti-laminin in rat sciatic nerve further revealed an additional selective distribution within the endoneurium. Under high magnification, C4S immunoreactivity appeared to surround each anti-laminin immunoreactive endoneurial tube while C6S labeling appeared to colocalize with laminin immunoreactivity (Fig 2).

### Perlecan colocalized with laminin and the ΔCS-C neoepitope in the endoneurium

**Fig 3:**
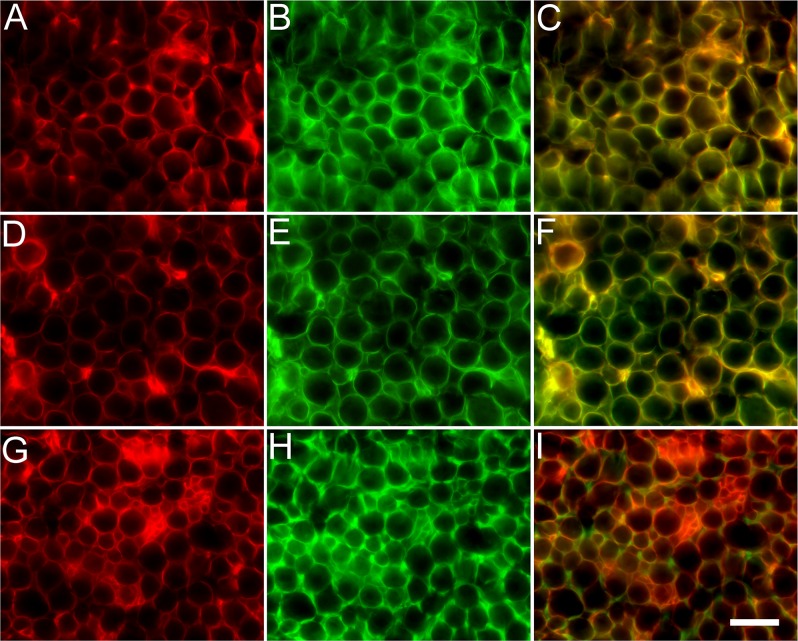
Perlecan distribution relative to laminin, ΔCS-A, and ΔCS-C in normal rat peripheral nerve. Normal rat sciatic nerve was subjected to chondroitinase ABC treatment and immunolabeled with antibodies against perlecan and laminin or ΔCS-C or ΔCS-A. (A) Perlecan was present within the endoneurium tubes and appeared to label the ring-like structures in a similar pattern to laminin (B). (C) Combination of filters revealed that both laminin and perlecan closely associate within the basal lamina tubes. (D) Perlecan also closely associated with ΔCS-C tubes (E) which was confirmed when filters were merged (F). (G) Perlecan did not colocalize with ΔCS-A (H) within the endoneurium and the combination of both filters clearly demonstrated a complementary distribution (I). Scale bar: 20 μm.

Perlecan is an integral component of the basal lamina tubes and colocalized with laminin (Fig 1A and 1B). Double immunolabeling for perlecan and laminin revealed an overlapping staining pattern of thin rings at higher magnification (Fig 3A, 3B and 3C). Following ChABC treatment, double labeling with antibodies against perlecan and ΔCS-C also revealed overlapping thin rings (Fig 3D, 3E and 3F). Meanwhile, double labeling with perlecan and ΔCS-A antibodies did not overlap, resulting in a complementary pattern where ΔCS-A appeared to surround the thin ringed profile of perlecan (Fig 3G, 3H and 3I). These results indicated that perlecan, laminin and ΔCS-C were integral components of the endoneurial basal lamina tubes while ΔCS-A surrounded the basal laminae (as shown in Fig 2).

### Optimum conditions for ChC treatment of nerve graft

**Fig 4:**
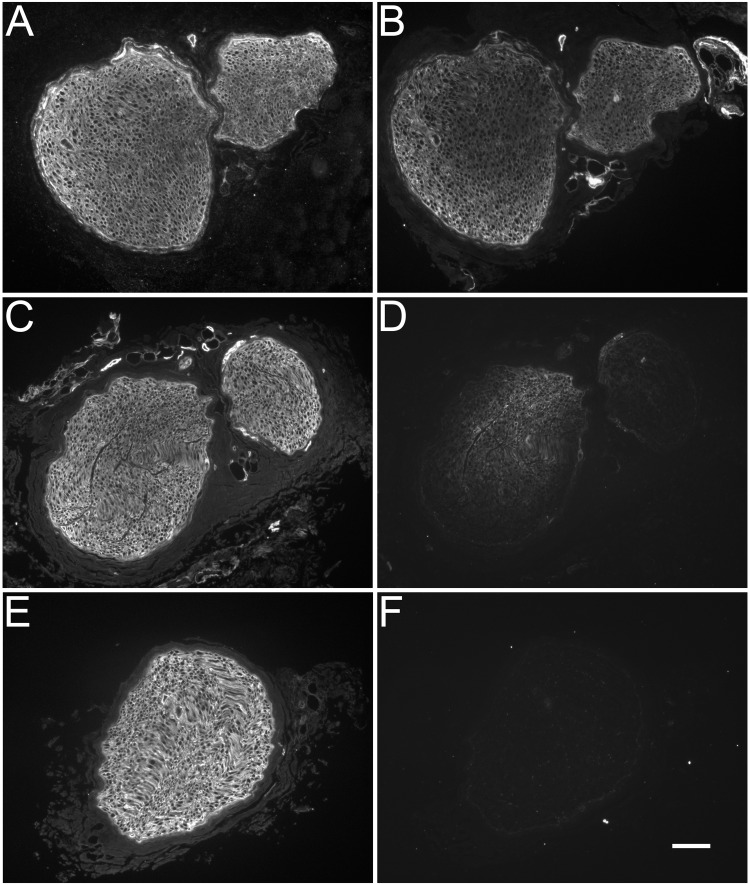
Optimum conditions for chondroitinase C treatment of nerve graft. Three-cm segments of rabbit peroneal nerve were subjected to freeze/thaw treatment and incubated in various concentrations of chondroitinase C at different time durations (1U/ml 24hours A and B; 4U/ml 16hours C and D; 4U/ml 24hours E and F). Samples were taken from the middle of the nerve segment and subjected to immunocytochemical analysis to determine which concentration/time duration revealed the C6S neoepitope (A, C, E) and completely removed the CS-56 immunoreactive glycosaminoglycan chains (B, D, F). (A) 1U/ml for 24 hours revealed the C6S neoepitope throughout the endoneurial tissue. (B) Serial tissue sections indicated that concentration was not effective at removing the CS-56 immunoreactive glycosaminoglycan chains. (C and D) 4U/ml for 16 hours incubation did not completely remove the CS-56 immunoreactive chains either. (E and F) 4 U/ml for 24 hours appeared to effectively remove the CS-56 immunoreactivity from within the endoneurial tissue. Scale bar: 100 μm.

The goal of these experiments was to develop a nerve graft that has been enzymatically treated with ChC. To achieve this, we modified a previously published method for complete degradation of CSPGs using ChABC [16] based on the enzymatic properties of ChC as first described by Michelacci and Dietrich (1976) [20]. Three-cm rabbit nerve segments were treated with various concentrations of ChC (2U, 4U, 8U per ml) for 4, 8, 16, and 24 hours, and then immunolabeled with antibodies C6S and CS-56. The results indicated that 4U/ml for 24 hours completely removed CS-56 immunoreactivity and produced the C6S neoepitope labeling. This indicated that ChC completely degraded the GAG chains from the endoneurial tissue of the nerve graft segment (Fig 4). Therefore, 4U/ml for 24 hours became the standard for all ChC treatments whether *en bloc* or directly on tissue slices.

### ChC treatment degrades endoneurial proteoglycans producing ΔCS-C

**Fig 5:**
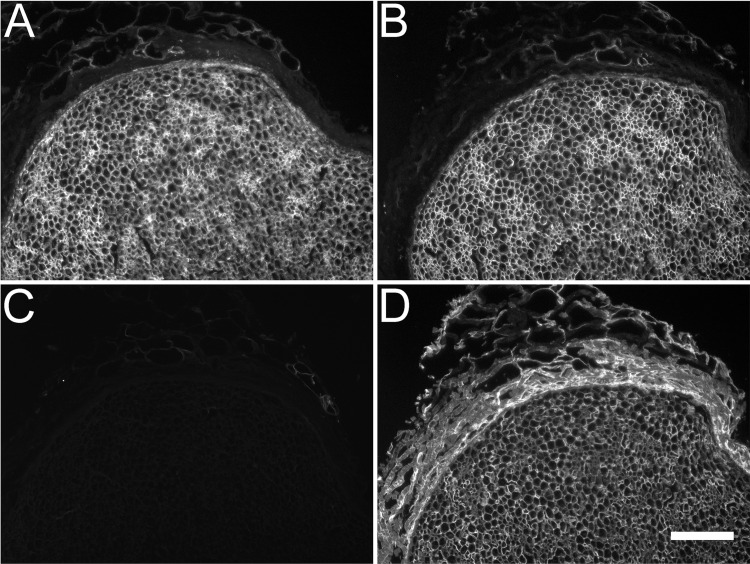
Chondroitinase C treatment on normal peripheral nerve reveals ΔCS-C only. Normal rabbit nerves were subjected to chondroitinase C treatment only (A and C) or by an additional treatment of chondroitinase ABC (B and D). Tissue was then labeled with ΔCS-C (A and B) or ΔCS-A (C and D) antibodies (C6S or C6S respectively). (A) C6S was present exclusively within the endoneurium of chondroitinase C treated nerve. (B) A secondary chondroitinase ABC treatment did not reveal any additional C6S immunoreactivity indicating that chondroitinase C effectively degraded the CS-C glycosaminoglycan chains. (C) Chondroitinase C treatment did not create ΔCS-A immunoreactivity, which provided evidence of substrate specificity. (D) A secondary chondroitinase ABC treatment did reveal the C4S neoepitope confirming that chondroitinase C is selective for CS-C glycosaminoglycan chains. Scale bar: 100μm.

**Fig 6:**
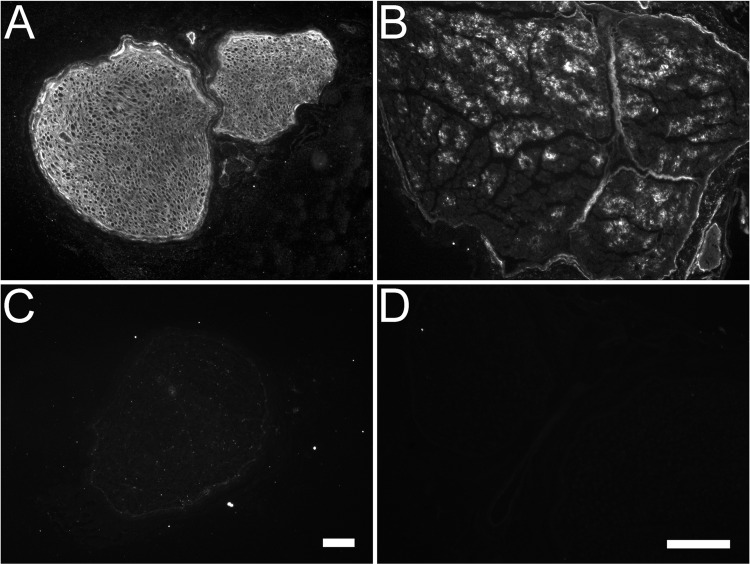
Chondroitinase C effectively removed endoneurial chondroitin sulfate glycosaminoglycans. Normal human (A and C) and normal rabbit (B and D) were treated with chondroitinase C (C and D) and immunolabeled for CS-56 (A and C) or 473-HD (B and D). (A) CS-56 was present throughout the normal human nerve endoneurium. (B) 473-HD immunolabeled a subset of endoneurial basal laminae tubes within the rabbit nerve. Chondroitinase C treatment effectively removed immunoreactivity of both CS-56 (C) and 473-HD (D) antibodies which indicated the glycosaminoglycan chains were cleaved off the core protein. Scale bar: 100μm.

Based on the distribution of ΔCS-A and ΔCS-C in the peripheral nerve, we postulated that ChC would selectively degrade the CS-C GAGs isolated to the basal lamina tubes within the endoneurium. To test this, peripheral nerve sections were treated with ChC and immunolabeled for ΔCS-C. As hypothesized, ChC treatment produced ΔCS-C in the endoneurium (Fig 5A). To ensure that ChC completely degraded all CS-C GAGs, sections pretreated with ChC were subjected to additional ChABC treatment, immunolabeled for ΔCS-C and visually compared. The secondary ChABC treatment did not reveal additional ΔCS-C immunoreactivity, which indicated that ChC effectively degraded all CS-C GAGs within the tissue section (Fig 5B). To test if ChC was selective for producing ΔCS-C, we immunolabeled for ΔCS-A after ChC treatment. The results showed that ChC treatment does not produce detectable ΔCS-A in nerve tissue (Fig 5C), while ChABC treatment did (Fig 5D). To determine which GAG chains were susceptible to ChC treatment, we immunolabeled normal (Fig 6A and 6B) and ChC treated nerve sections (Fig 6C and 6D) with CS-56 and 473-HD antibodies which recognize intact CS GAG chains. Results indicated that both CS-56 and 473-HD immunoreactive GAG chains were present within the endoneurium of normal nerve and that ChC treatment removed these GAG chains resulting the absence of immunoreactivity.

### ChC treatment selectively enhances the growth-promoting property of the endoneurium

We provide evidence that ChC degrades CS-56 and 473-HD immunoreactive GAG chains within the endoneurium and produces ΔCS-C neoepitope. To test if there is a functional consequence of ChC treatment, we performed cryoculture bioassays on fresh frozen rabbit nerve tissue sections seeded with neonatal mouse DRG neurons (see Methods and Materials). Nerve sections were pretreated with ChABC, ChC, or a vehicle control. Neurites were visualized; lengths were measured and then categorized as either growing in the endoneurial or epineurial tissue (S1 Table). ChABC and ChC treatments increased the lengths of neurites growing on the endoneurium by 81% compared to the vehicle control treatment (see Table 2). ChABC treatment also increased the length of neurites growing in the epineurial tissue by 87% compared to both ChC and vehicle treatments. These results provide evidence that ChC treatment selectively enhanced the growth-promoting property of the endoneurium while retained the growth-inhibiting property of the epineurium.

**Table 2:** Cryoculture results.

Condition	Endoneurium^a^	Epineurium^a^
ChABC	*551.6μm ± 27.3; n = 44	**275.3μm ± 18.9; n = 22
ChC	*538.1μm ± 40.9; n = 35	146.7μm ± 11.6; n = 10
Vehicle	*304.9μm ± 23.0; n = 46	153.0μm ± 33.7; n = 8

^a^ Mean axon length ± standard error of the mean; n

*Considered statistically significant compared to vehicle treatment. p<0.001

**Considered statistically significant compared to vehicle and ChC treatment. p<0.001

**Fig 7:**
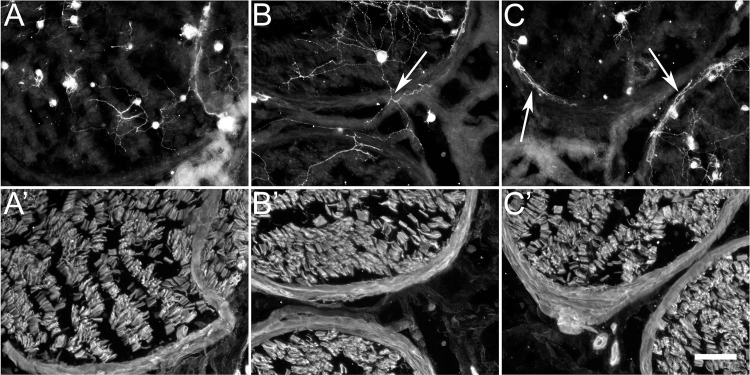
Cryoculture demonstrates differential functional effects from chondroitinase ABC and C treated nerve tissue. Normal human nerve was tranversely sectioned and treated with vehicle control (A and A’), ChABC (B and B’), or chondroitinase C (C and C’) and subjected to cryoculture bioassay. Cultures were double immunolabeled for Gap43 (A, B, C) and laminin (A’, B’,C’). (A) Vehicle treated tissue sections provided a poor substrate for neurite extention either within or outside the nerve fascicles. (B) ChABC treated tissue significantly enhanced the ability for neurite extension and arborization within the nerve fascicles. Furthermore, neurites readily cross the perineurial and epineurial tissue to extend neurites from one fascicle to another (B arrow). (C) ChC treatment moderately improved neuritic growth throughout the endoneurium. Interestingly, neurites did not cross the perineurium surrounding the fascicles (C arrows) indicating that ChC selectively removed endoneurial inhibitory proteoglycan glycosaminoglycan chains while retaining the inhibitory properties of the perineurium and epineurium. (A’, B’, C’) Laminin immunolabeled tissue demarkated nerve fascicles. Scale bar: 100μm.

The perineurium is a nerve sheath that surrounds each fascicle and serves as a boundary between endoneurium and epineurium. To determine if ChC altered the growth-promoting property of the perineurium, we performed cryoculture assays on transverse sections of human nerve and double labeled for GAP43 and laminin antibodies (Fig 7). Anti-GAP43 labels growing neurites and laminin immunolabeling was used to define the nerve sheaths and fascicle boundaries. As expected, vehicle treated nerve sections provided a poor substratum for neurite growth both inside and outside the nerve fascicle (Fig 7A). ChABC treatment greatly enhanced neuritic growth associated with endoneurial tubes (Fig 7B). Additionally, neurites crossed from one nerve fascicle to another, which required growth cones to migrate across the perineurium and epineurium in between fascicles. ChC treatment provided an intermediately permissive substrate where neurites grew well within the endoneurium but generally did not cross the surrounding perineurium (Fig 7). Instead, neurites growing on the ChC treated nerve section mainly turned and grew along the interface of the perineurial sheath. This provided additional evidence that degradation of CS-C GAGs by ChC treatment enhanced the growth-promoting property of the endoneurium without inactivating the inhibitory GAG chains of the perineurium and epineurium.

### ChC treatment selectively degrades GAG chains from particular CSPGs

**Fig 8:**
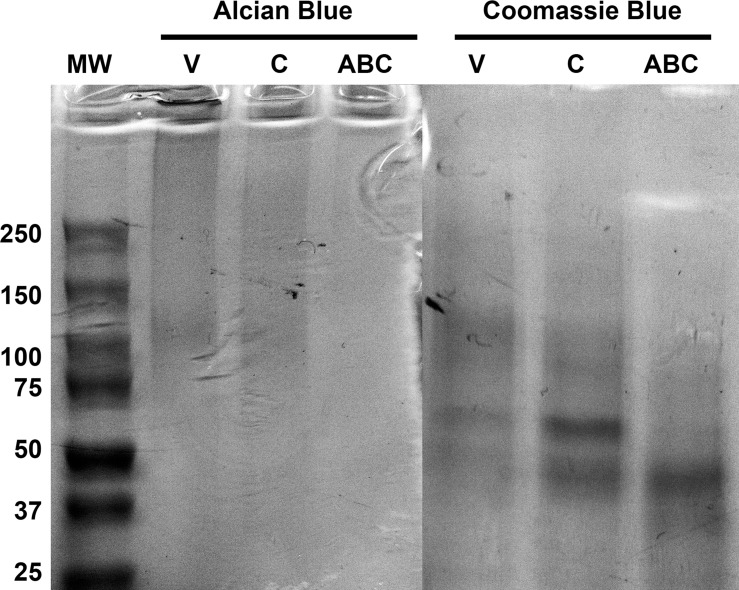
SDS-PAGE analysis of normal rabbit nerve proteoglycan extraction with Alcian Blue and Coomassie Blue. The proteoglycan content of 40 normal rabbit sciatic nerves were extracted and isolated through DAEA column chromatography. Samples were treated with vehicle buffer (V), chondroitinase C (C), or chondroitinase ABC (ABC) and subjected to 4–15% SDS-PAGE separation. Gels were then stained for Alcian Blue or Coomassie Blue to label glycosaminoglycans or protein content respectively. Vehicle treated samples resulted in a broad band when stained with Alcian Blue which indicated the presence of glycosaminoglycan chains of various molecular masses including three bands at the top of the gel interface, a broad band corresponding to M_r_ = 300-450kD, and band between 100 and 130kD. Coomassie Blue staining of the corresponding column also visualized several broad bands at lower molecular weight including several strong bands between M_r_ = 100-130kD, 65kD, and 45-55kD. Chondroitinase C treated samples removed the Alcian Blue stained bands at the gel interface but retained the bands between M_r_ = 300-450kD and 100-130kD. Coomassie Blue staining provided evidence of increased intensity of the M_r_ = 65kD and 45-55kD band. Chondroitinase ABC treated samples were devoid of Alcain Blue staining which indicated the absence of glycosaminoglycans. Coomassie Blue staining showed a reduction in staining intensity for the M_r_ = 100-130kD and 65kD band while the 45-55kD band shifted downward to aproximately 35-45kD.

We found that ChC effectively and selectively degrades CS GAGs within the endoneurium. To identify proteoglycans that contain the ChC -sensitive GAG chains, we examined proteoglycan-enriched nerve extract (PENE) from normal rabbit nerve pretreated with ChABC, ChC, or vehicle control by SDS-PAGE and Western immunoblotting. Alcian Blue stains intact GAG chains present in SDS-PAGE [33]. Vehicle treated PENE produced a broad diffuse Alcian Blue stained smear from the gel interface to about M_r_ = 50kD (Fig 8). Three darker bands appeared within the column with one at the top of the gel, a broad band from M_r_ = 300-450kD and a band between 100 and 130kD. ChC treatment eliminated the band at the gel interface and reduced the intensity of the M_r_ = 300-450kD band, but did not alter the M_r_ = 100-130kD band. ChABC treatment completely removed all GAGs as indicated by the lack of detectable Alcian Blue stain.

Coomassie Blue stains protein content within a SDS-PAGE and therefore the proteoglycan core proteins [34]. Coomassie Blue barely detected protein bands that corresponded to the M_r_ = 300-450kD bands stained by Alcian Blue but did reveal several protein bands at 100-130kD, 65kD, 45-55kD, and 25kD in the vehicle treated samples. ChC treatment increased the stain intensity of the M_r_ = 65kD and 45-55kD bands. ChABC significantly reduced the intensity of the M_r_ = 100-130kD and 65kD bands and shifted the 45-55kD band to 35-45kD. This indicated that ChC affected some of the massive proteoglycans but not the lower M_r_ = subtypes observed in the vehicle treated samples. As expected, altogether, ChABC significantly decreased the mass or dispersed the majority of the proteoglycan bands.

**Fig 9:**
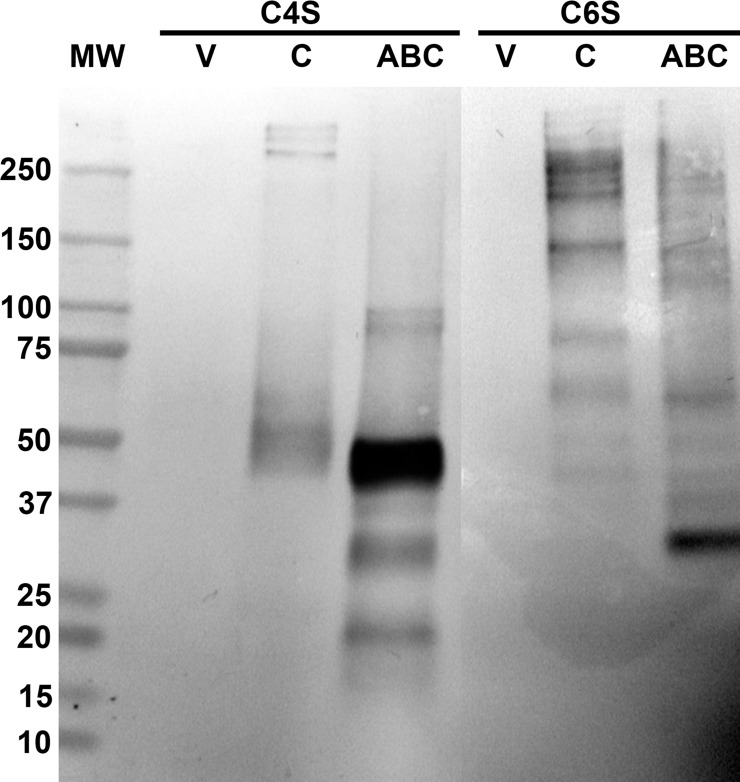
Western analysis of neoepitope immunolabeling of enzyme or vehicle treated proteoglycan extract. Proteoglycan enriched nerve extract (PENE) was obtained as described in Methods and Materials and were pretreated with vehicle buffer (V), chondroitinase C (C), or chondroitinase ABC (ABC) prior to 4–15% SDS-PAGE separation and transferred to nitrocellulose for western analysis with neoepitope antibodies. Vehicle treated samples did not reveal any immunoreactivity as expected. Chondroitinase C and chondroitinase ABC treated PENE produced differential immunoreactive band indicating selective degradation of glycosaminoglycans and the production of ΔCS-A and ΔCS-C. Surprisingly, Chondroitinase C treated PENE was immunoreactive for C4S producing three distinct bands of high molecular mass and a broad band at M_r_ = 45-55kD. Chondroitinase ABC eliminated the high molecular weight C4S bands, created a doublet at M_r_ = 100kD, and several lower mass bands including a very intense band at approximately 45kD. C6S labeling produced several bands for chondroitinase C treated PENE. Chondroitinase ABC treated PENE produced several identical C6S immunoreactive bands as observed in chondroitinase C treated PENE with a few exceptions including the elimination of the 75kD band and the creation of a 30kD band.

Because ChABC and ChC treatment produces residual ΔCS-A and/or ΔCS-C that are attached to the proteoglycan core protein, shifts in the mass of chondroitinase-sensitive proteoglycans can be observed by C4S and C6S neoepitope immunolabeling on Western blots. Western immunoblotting of ChABC and ChC treated PENE revealed the creation of several different CSPG variants that were labeled by C4S and C6S antibodies (Fig 9). ChABC treatment produced a C4S immunoreactive doublet at M_r_ = 90-100kD, a major band at 45kD, a small isolated band at 37kD and two intense bands at 30kD and 20kD. Interestingly, ChC treated samples produced isolated C4S immunoreactive bands of high MW (>250kD) and a lower mass cluster around 50kD. This indicated that ChC could produce ΔCS-A even though immunohistochemical methods may not have been sensitive enough to detect it in nerve tissue sections. ChC treatment produced a C6S immunoreactive cluster around M_r_ = 250kD and bands at 150kD, 95kD and 65kD. ChABC treatment reduced the intensity of the 250kD, 150kD, and 95kD bands observed in ChC treated samples and produced several smaller mass C6S immunoreactive bands at 140kD, 130kD, 65kD, and between 50-30kD. Vehicle treated samples did not react with either neoepitope antibodies. These results indicate that ChC treatment of PENE selectively degraded some CS GAG chains producing higher M_r_ proteoglycan species while ChABC treatment degraded all CS GAG chains as evident by the loss of higher mass proteoglycans.

### Lower molecular weight CSPGs are not degraded by ChC

**Fig 10:**
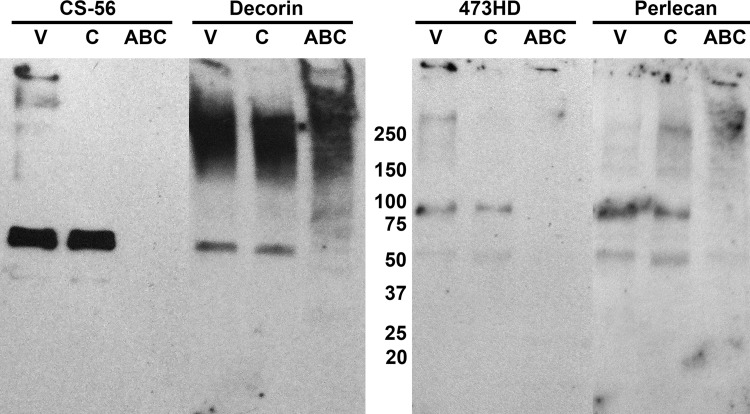
Identification of proteoglycan core proteins and glycosaminoglycan chains in western analysis of enzyme or vehicle treated proteoglycan extract. Proteoglycan enriched nerve extract (PENE) was obtained as described in Methods and Materials. Western analysis was performed using core protein antibodies against decorin and perlecan as well as the glycosaminoglycan antibodies CS-56 and 473-HD. Vehicle (V) treated PENE provided various immunoreactive bands of different molecular weight against CS-56 and 473-HD. Chondroitinase C (C) treatment eliminated all the higher molecular weight bands of CS-56 but retained a CS-56 immunoreactive doublet at M_r_ = 60-65kD and a faint band at 45kD that was observed in the vehicle treated PENE. Meanwhile, chondroitinase ABC (ABC) treatment completely eliminated CS-56 immunoreactivity from the PENE. Decorin appeared as a very broad band between M_r_ = 140-350kD and an isolated 60kD band in the vehicle treated sample. Chondroitinase C treated PENE did not provide remarkable changes in the decorin immunoreactive bands but chondroitinase ABC modified the immunoreactivity slightly by creating a few bands at 90kD and 70KD while eliminating the 60kD band. 473-HD immunoreactivity was observed in several bands at 300kD, 90kD, 55kD, and at the gel interface of the vehicle treated PENE while chondroitinase C treatment reduced the intensity of the higher weight bands but retained the 90kD and 55kD bands. Chondroitinase ABC treated PENE did not contain any 473-HD immunoreactivity. Perlecan antibody bound to several bands at M_r_ = 250kD, 165kD, and 155kD as well as a strong band at 90kD and a weak band at 55kD in the vehicle treated PENE. Chondroitinase C treated PENE increased the intensity of perlecan immunoreactivity in the M_r_ = 250kD band while reducing the intensity of the 165kD and 155kD but retained the 90kD and 55kD band. Interestingly, chondroitinase ABC treatment did not modify the higher molecular weight bands of perlecan but did remove the 90kD and 55kD bands.

Based on the western analysis results of C4S and C6S immunoreactivity, we wanted to identify particular proteoglycan core proteins and GAG chains that were sensitive to ChABC and ChC treatments. Western blots were labeled with antibodies against perlecan, versican, decorin, and the GAG antibodies CS-56 and 473-HD (Fig 10). Immunodetection of versican was poor and difficult to interpret. CS-56 immunoreactivity in the vehicle treated PENE revealed bands with M_r_ = 300-450kD, 250kD, a doublet at 60-65kD, and a weaker band at 45kD. Furthermore, there was significant immunoreactivity at the gel interface that indicated some undigested proteoglycans were too large to enter the gel (despite highly dissociating and reducing conditions). ChC treated PENE lacked the higher mass CS-56 immunoreactive bands including the band at the gel interface but retained the doublet at 65kD and the band at 45kD. No CS-56 immunoreactivity was observed when the nerve extract was pretreated with ChABC, confirming the Alcian Blue findings that all CS GAG chains were enzymatically degraded. The decorin antibody labeled a broad smear between 140-350kD and a 60kD band in the vehicle treated PENE. Interestingly, ChC treated PENE had the same decorin immunoreactive profile as the vehicle treated PENE. ChABC treated samples also contained the 140-350kD immunoreactive smear, dispersed the 60kD band and produced 2 additional bands at 90kD and 70kD. 473HD immunoreactivity was observed at the gel interface, at 300kD, 90kD, and a light band at 55kD in the vehicle treated PENE. After ChC treatment, the 473HD immunoreactive band at the gel interface was dispersed along with the band at 300kD, while the 90kD and 55kD bands were not altered. ChABC treatment eliminated all of the 473HD immunoreactive bands identified in both ChC and vehicle treated PENE. The perlecan antibody weakly labeled three high mass bands at M_r_ = 250kD, 165kD and 155kD, a stronger band at 90kD and a band at 55kD in the vehicle treated PENE. ChC treatment increased the intensity of the 250kD band, while the 165kD and 155kD bands appeared more diffuse and the 90kD and 55kD band were unchanged. ChABC treated PENE produced a perlecan immunoreactive smear around 250kD band and 160kD but eliminated the 90kD and 55kD bands. These results provided evidence that ChC selectively degraded CS-56 and 473HD immunoreactive GAG chains associated with high mass fragments of unidentified proteoglycans but not the lower mass fragments of perlecan and decorin.

## Discussion

The enzymatic activities of ChABC are well characterized. This lyase is known to fully degrade CS GAG and DS GAG chains to their unsaturated disaccharide subunits through beta-eliminase activity of the β1–4 glycosidic bond through both endolytic and exolytic mechanisms [35]. When this reaction occurs at the tetrasaccharide linkage region adjacent to the core protein, the initial GAG subunit is retained forming an unsaturated hexuronic acid that is recognized by C4S and C6S antibodies [28] [31]. It is important to note that these antibodies will only describe the residual unsaturated GAG unit after ChABC treatment and cannot directly determine the composition of the CS GAG chains. Meanwhile, CS-56 and 473HD antibodies recognize a specific sequence of CS GAG subunits that require a disulfated CS-D subunit for effective binding [26]. We combined the use of both neoepitope and native GAG antibodies to expand our understanding of the distribution of CSPG types in the peripheral nerve. Based on our results, we surmised that the GAG chains of the endoneurium of normal peripheral nerve must contain a CS-D subunit due to the tissue specificity of both CS-56 and 473HD immunoreactivity. Furthermore, CS-56 is associated with the entire endoneurium while 473HD labels a subset of endoneurial tubes. Additionally, proteoglycans associated with the endoneurial basal laminae tubes have GAG chains that have an initial ΔCS-C unit attached to the tetrasaccharide linkage region while the remainder of the interstitial tissues (within the endoneurium or epineurium) have an initial ΔCS-A unit.

Since the distribution of permissive (laminin) and inhibitory (CSPGs) components are postulated to produce an ECM that suppresses spontaneous neurite sprouting and maintain homeostasis in normal peripheral nerve, we were not surprised to observe a complementary distribution of ΔCS-A and ΔCS-C within the endoneurial tissue. We found that laminin, ΔCS-C, and perlecan colocalized on the basal laminae tubes of the endoneurium along with CS-56 and 473HD immunoreactive GAG chains in normal peripheral nerve. Meanwhile, ΔCS-A appeared only in the interstitial tissue surrounding the basal laminae tubes. Perlecan is considered a hybrid proteoglycan containing both CS and heparan sulfate chains which have been shown to be neutral or slightly permissive to neurite sprouting in culture [6]. CS56 immunoreactive GAG chains are upregulated following injury in the spinal cord and are considered inhibitory towards axon regeneration [36] [37], while 473HD immunoreactive GAG chains have been identified as a neurite promoter of hippocampal neurons in culture [38] [39]. Interestingly, during CSPG synthesis, 4-sulfation of the initial CS GAG unit has been associated with GAG chains containing mostly CS-A [40]. Furthermore, CS-A GAG chains have been shown to have strong inhibitory activities against neurite formation [3] [41]. Meanwhile, chondroitin 6-sulfotransferase 1 expression, an enzyme responsible for the addition of sulfate groups on the C-6 position during GAG synthesis, has been found to be highly expressed in Neu7 and NG2 astrocytes. Additionally, CS-C concentrations were found to be increased and may contribute to the inhibitory nature of the glial scar following cortical stab injuries [42].

Taken together, we propose that the basal laminae tubes contain permissive laminin and neutral perlecan with inhibitory CS-56 and permissive 473HD GAG chains, surrounded by inhibitory GAG chains that are immunoreactive to C4S following ChABC treatment. This would produce a generally permissive substrate that is modulated by inhibitory and permissive GAG chains that could serve as an additional mechanism for maintaining axonal homeostasis. Furthermore, since both CSPGs and laminins are upregulated after nerve injury [7], this distribution may also provide a reasonable explanation for successful regeneration following a nerve crush injury in which the endoneurial structure is not disrupted. Regenerating axons would sprout and extend neurites within the endoneurial tube isolated from the inhibitory CS-A GAG chains surrounding each tube. Meanwhile, a nerve transection or the implantation of a nerve graft would cause disruption in the basal lamina tube structure where sprouting axons could be in direct contact with inhibitory CS-A GAG chains surrounding the endoneurial tubes or to a larger extent, CS-A GAG chains found outside the nerve fascicles. Interestingly, application of ChABC significantly enhanced axon regeneration following a nerve transection but not a nerve crush injury [43].

The enzymatic activity of ChC remains somewhat enigmatic. So far we understand that ChC enzymatically cleaves the β1–4 glycosidic bond between the NGalAc (regardless of sulfation) of one GAG unit and the preceding CS-C GAG unit in an endolytic method [32]. ChC also can cleave, albeit with less efficacy, the β1–4 glycosidic bond if the preceding GAG unit is a CS-A GAG, but not at all if the preceding GAG chain is a DS-GAG [4] [20]. Our results indicate that: 1) ChC has the ability to cleave CS-56 and 473HD immunoreactive GAG chains in nerve tissues. 2) ChC treatment creates ΔCS-C isolated to the nerve fascicle endoneurium but not within the surrounding interstitial tissue of the endoneurium or epineurium. 3) ChC treatment selectively enhanced the neurite growth promoting properties of the nerve endoneurium as shown with the cryoculture bioassay. 4) ChC treatment on PENE will produce mostly C6S immunoreactive bands and limited C4S immunoreactive bands with high mass. 5) ChC treatment degraded some of the CS GAG chains of perlecan but did not degrade the CS/DS GAG chains of decorin. We provided evidence for selective degradation of proteoglycan GAG chains through western analysis of PENE and immunocytochemical analysis on nerve tissue sections. Furthermore, selective degradation provided meaningful functional consequences in neurite extension using cryoculture.

There are some limitations to the results presented here. The ChABC product used in our studies has been shown to retain the initial GAG subunit on the tetrasaccharide linkage region while ChABC from another source was reported to remove the initial GAG subunit [44]. Secondly, ChC has been reported to can degrade the initial GAG subunit depending on the sulfation substitution within the tetrasaccharide linkage area [45]. Theoretically, this could complicate the interpretation of both C4S and C6S immunolabeling by removing the ΔCS-A and ΔCS-C antigen and providing a false negative. To address these concerns, we performed C6S and C4S immunolabeling on tissue sections pretreated with ChABC or ChC as well as a sequential application of ChABC after ChC treatment to test ChC efficacy (Fig 5). There were no remarkable differences of neoepitope labeling or distribution in the tissue following serial treatments of ChABC or ChC. Furthermore, cryoculture results indicate that ChC treatment selectively enhanced the regenerative capacity of the tissue that corresponded to C6S immunoreactivity, namely the endoneurial tissue.

Normal peripheral nerve contains a balance of inhibitors and promoters for axon regeneration. The distribution of CSPG and laminin in normal nerve provides an overall inhibitory environment and complete removal of GAG chains through ChABC treatment may increase axon sprouting and provide an uncontrolled environment that could lead to increased aberrant regeneration outside the nerve fascicles. ChC treatment selectively enhanced the regenerative capacity of the endoneurium while retaining the inhibitory properties of the perineurium and epineurium. The novel findings in this report are a solid foundation for additional investigations of the in vivo effects of ChC treatment of both peripheral and central nervous tissue.

## Supporting Information

S1 Table Raw data from cryoculture analysis. Cryoculture axon lengths were measured from the neuron cell body towards the longest neurite. Measurements were congregated based on endoneurial or epineurial growth. Measurements, mean values, standard deviations and standard error of the means were reported as μm.
(TIF)

## Abbreviations

CSPG: Chondroitin sulfate proteoglycan
ChABC: chondroitinase ABC
ChC: chondroitinase C
GAG: glycosaminoglycans
ECM: extracellular matrix
NGalAc: N-acetylgalactosamine
GlcA: glucuronic Acid
IdoA: iduronic acid
ΔCS-A: unsaturated chondroitin sulfate A
ΔCS-C: unsaturated chondroitin sulfate C
