# Supplementary figures and images for: Chondroitinase C Selectively Degrades Chondroitin Sulfate Glycosaminoglycans that Inhibit Axonal Growth within the Endoneurium of Peripheral Nerve
Source: PLoS One. 2016 Dec 14;11(12):e0167682. doi: 10.1371/journal.pone.0167682 (PMC5156433; doi:10.1371/journal.pone.0167682)

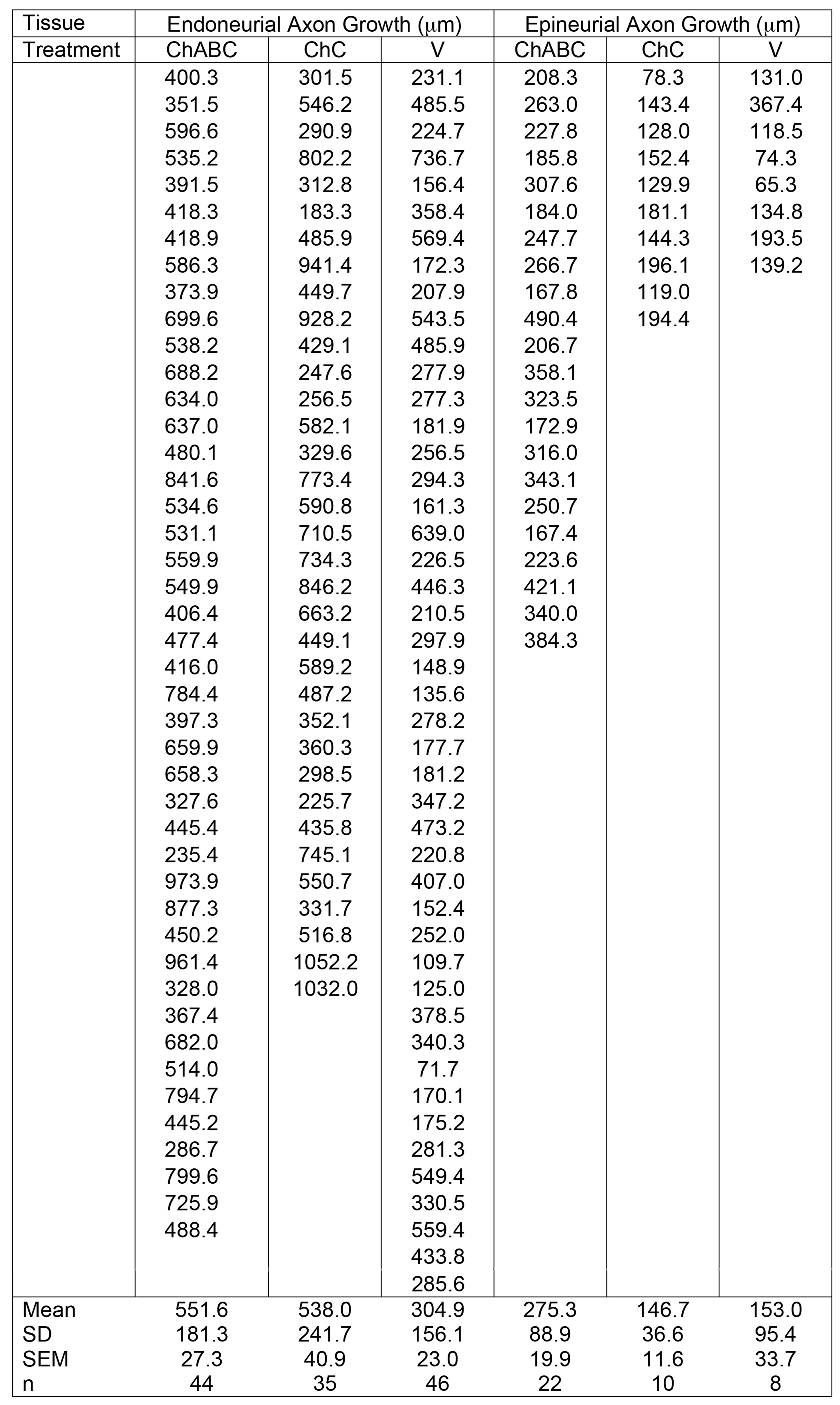

Supplement: S1 Table — Cryoculture axon lengths were measured from the neuron cell body towards the longest neurite. Measurements were congregated based on endoneurial or epineurial growth. Measurements, mean values, standard deviations and standard error of the means were reported as μm. (TIF) [file pone.0167682.s001.tif]
